# 3′UTR SL-IV and DB1 Regions Contribute to Japanese Encephalitis Virus Replication and Pathogenicity

**DOI:** 10.3389/fvets.2021.703147

**Published:** 2021-08-02

**Authors:** Jinchao Xing, Youyue Zhang, Ziying Lin, Lele Liu, Qiang Xu, Jiaqi Liang, Zhaoxia Yuan, Cuiqin Huang, Ming Liao, Wenbao Qi

**Affiliations:** ^1^Key Laboratory of Zoonoses, Ministry of Agriculture and Rural Affairs, South China Agricultural University, Guangzhou, China; ^2^National and Regional Joint Engineering Laboratory for Medicament of Zoonoses Prevention and Control, Guangzhou, China; ^3^Guangdong Laboratory for Lingnan Modern Agriculture, Guangzhou, China; ^4^College of Animal Sciences and Technology, Zhongkai University of Agriculture and Engineering, Guangzhou, China; ^5^The Key Laboratory of Fujian Animal Diseases Control, Longyan University, Longyan, China; ^6^Key Laboratory of Zoonoses Prevention and Control of Guangdong Province, Guangzhou, China

**Keywords:** Japanese encephalitis virus, 3′ untranslated region, stem-loop IV, virus replication, pathogenicity

## Abstract

Japanese encephalitis virus (JEV), a mosquito-borne flavivirus that causes fatal neurological disease in humans, is one of the most important emerging pathogens of public health significance. JEV is maintained in an enzootic cycle and causes reproductive failure in pigs. Notably, the shift in JEV genotypes is not fully protected by existing vaccines, so the development of a candidate vaccine is urgently needed. In this study, we compared pathogenicity between Japanese encephalitis virus SA14 and BJB (isolated from humans in the 1970s) strains. We found that the BJB strain was attenuated in mice and that there was no case fatality rate. The growth rate of BJB was higher than SA14 virus in BHK-21 cells. Based on the sequence alignment of the viral genome between the SA14 and BJB virus strains, some mutations at sites 248, 254, 258, and 307 were observed in the 3′ untranslated region (3′UTR). The 3′UTR of JEV plays a very important role in the viral life cycle. Furthermore, using a reverse genetic system, we conducted and rescued the parental JEV strain SA14 (T248, A254, and A258) and the mutant virus rSA14-3′UTRmut (T248C, A254G, A258G, and 307G). Through an analysis of the RNA secondary structure model of the 3′UTR, we discovered that the mutations of T248C, A254G, and A258G reduced the apiculus ring and increased the lateral ring significantly in the stem-loop structures IV (SL-IV) structure region of 3′UTR. Moreover, the insertion of 307G added a ring to the dumbbell structure 1 (DB1) structure region. Strikingly, these RNA secondary structure changes in 3′UTR of rSA14-3′UTRmut increased viral negative chain RNA production and enhanced the replication ability of the virus in BHK-21 cells. However, *in vivo* mouse experiments illustrated that the rSA14-3′UTRmut virus significantly decreased the neurovirulence of JEV. These results affirmed that the JEV SL-IV and DB1 regions play an important role in viral proliferation and pathogenicity. Taken together, we complement the study of RNA element function in the 3′UTR region of JEV by providing a new target for the rational design of live attenuated candidate vaccines and the increase of virus production.

## Introduction

Japanese encephalitis virus (JEV) belongs to the genus *Flavivirus* in the family *Flaviviridae*. Flaviviruses such as dengue virus (DENV), Zika virus (ZIKV), and West Nile virus (WNV) can cause viral encephalitis, Guillain-Barré syndrome, and infant microcephaly (1). JEV is maintained in an enzootic cycle involving mosquito vectors (particularly *Culex tritaeniorhynchus*) and amplified in the main vertebrate of host pigs and wading birds (2, 3). The JEV exists as five distinguishable genotypes (G-I, G-II, G-III, G-IV, and G-V) based on nucleotide homology in the E protein gene (4, 5). JEV G-III was the historically dominant genotype throughout most of Asia, but it has been gradually replaced over the last 20 years by G-I in many Asian countries (6, 7). However, the shift in JEV genotypes is not fully protected by existing vaccines (8, 9). The need for new JE vaccine strategies is urgent.

The JEV genome is a single-stranded, positive-sense RNA about 11 kb in length, and it encodes a single polyprotein that is processed post-translationally into structural and non-structural proteins by cellular and viral proteases (10). The structural proteins make up the virion (11), and the non-structural proteins have multiple functions during the virus life cycle, including virus replication and host immune evasion (12–14). Significantly, highly conserved secondary structures are formed by the 5′ and 3′ untranslated regions (UTR) and are implicated in virus replication, translation and packaging of the genome (15).

Recently, studies on flaviviruses have shown that the *cis*-RNA structural elements of the untranslated region of the flavivirus play an important role in immune escape and virus replication in host cells (16–18). For the synthesis of the genomic RNA to take place, the replicase complex must specifically recognize viral *cis*-acting RNA elements, defined by primary sequences or secondary/tertiary structures (19). These RNA elements are found in various locations within the genome but most frequently are located in the 5′ and 3′UTRs (17). The hairpin structure of 3′UTR is specific to the host for DENV, and the point mutation in the hairpin structure eliminated infection by the virus in mosquito cells but did not affect the replication of the virus in mammalian cells (20, 21). In vertebrate and invertebrate cells infected with DENV, the restriction of host cells results in selective genetic mutation of the virus, and the virus 3′UTR often has a hot spot for mutation (22, 23). The 3′UTR includes four stem-loop structures (SL, named SLI, SLII, SLIII, and SLIV) and two dumbbell structures (DB, named DB1 and DB2). SL and DB structures are essential in the initial virus replication process. SL-II structures are the main components that prevent 5′-3′ RNA Xrn1 from degrading and that produce subgenomic flavivirus RNA (sfRNA) (24). This sfRNA plays an important role in causing cell lesions and pathogenicity by participating in the apoptosis of host cells and regulating the host's antiviral type I interferon response (25–27). The growth efficiency of DENV which deleted DB1 or DB2 was reduced (28). In addition, while deletion of DB1 reduced replication of DENV, viruses lacking DB2 displayed a great increase of fitness in mosquitoes (29). Although research on flavivirus UTR has been carried out in depth, the current studies mainly focus on the RNA elements SL-II and DB2 in the 3′UTR. Research on the RNA element SL-IV and DB1 structures of JEV is still rare.

In this study, we found that the BJB strain, isolated from human in the 1970s, was attenuated in mice, and that there was no case fatality rate. Based on the sequence alignment of the viral genome, we found some mutations at sites 248, 254, and 258, as well as an insert at site 307 in the 3′UTR. Through analysis of the RNA secondary structure model of the 3′UTR, it was found that the nucleotide mutations of rSA14-3′UTRmut caused the top loop to shrink and the side loop to increase in the SL-IV structure region, and a ring was added to the DB1 structure region. These RNA secondary structure changes enhanced the replication of the virus in BHK-21 cells and significantly reduced pathogenicity in mice as well as the ability of neuron invasion. Collectively, this study provides a new perspective for the weakening of candidate vaccine strains and the increase of virus production.

## Materials and Methods

### Cells and Viruses

Baby hamster kidney cells (BHK-21) were maintained in Dulbecco's modified Eagle's medium (DMEM; Gibco), supplemented with 5% fetal bovine serum (FBS; Biological Industries, Israel) at 37°C in 5% CO_2_. The Japanese encephalitis virus (JEV) strains SA14 (pACYC-SA14, U14163) and BJB were kindly provided separately by Dr. Bo Zhang (Wuhan Institute of Virology, Chinese Academy of Sciences, China) and Dr. Changwen Ke (Guangdong Center for Disease Control and Prevention).

### Plasmid Construction

An infectious cDNA clone of JEV (pACYC-SA14) was used as the parental JEV strain for mutations. The mutant fragment was synthesized by the GENEWIZ company. The infectious cDNA clones pACYC-rSA14-3′UTRmut were handled using *Xba*I and *Xho*I sites to clone on the vector, pACYC. The following primers were used (5′-3′): 3′UTR -F:GGGTCATCTAGTGTGATTTAAGGTAGAAAA, 3′UTR -R: CCGACCCAGATCTTGTGTTCTTCCTCACCA, HDVr-F: ACTAGGCACAGAGCGCCGAAGTA, *Xho*I-R: ATTCAACGGGAAACGTCTTGCTCGA, *Xba*I-F: CATTTGGTTCATGTGGCTTGGAGCACGGTA, NS5-R: CTTAAATCACACTAGATGACCCTGTCTTCC.

### RNA Transcription *in vitro* and Transfection

The plasmids for the infectious clone were linearized using *Xho*I, followed in purification by phenol-chloroform extraction. RNA was then transcribed from the linearized plasmids using a T7 mMESSAGE mMACHINE kit (Ambion) according to the manufacturer's instructions. BHK-21 cells in 12- well plates were transfected with *vitro*-transcribed RNA using the Lipofectamine 2000 transfection reagent (Invitrogen) according to the manufacturer's instructions.

### Virus Replication in BHK-21 Cells

BHK-21 cells were infected with viruses at a multiplicity of infection (MOI) of 0.01 for 1 h and washed twice with phosphate-buffered saline (PBS) (Gibco), followed by the addition of medium. At 24, 48, and 72 h post-infection (hpi), the medium was harvested, and virus titer was determined in the BHK-21 cells.

### Virus Titer by TCID_50_ Method

Confluent monolayers of BHK-21 cells on 12-well plates were infected with wild-type and recombinant JEV and harvested at different time points post-infection. BHK-21 cells on 96-well plates were infected with 10-fold serial dilutions of the viral stock. 2 days post-inoculation, the infection situation of cells was examined by immunocytochemistry. BHK-21 cells on 96-well plates infected with JEV were washed three times with PBS and fixed with pre-cold methanol for 30 min at −20°C. Then, the cells were washed three times with PBS, incubated with primary antibody NS1 (ascitic fluid secreting mouse monoclonal anti-JEV NS1 by hybridoma cells) for 1.5 h at room temperature (RT), and followed by the appropriate secondary antibody HRP (Jackson ImmunoResearch, 115-035-003) for 1 h at RT. Finally, the cells were chemically stained with preparation mixture (1mL 3-Amino-9-ethylcarbazole solution (0.4g 3-Amino-9-ethylcarbazol powder, 100 mL Dimethylformamide), 15 mL 0.1 mol NaAc, and 0.15 mL 3% H_2_O_2_) for 30 min at RT and the number of infected wells were then counted using a microscope. The 50% tissue culture infection dose (TCID_50_) of each sample was determined using the Reed and Muench method.

### Real-Time Reverse Transcription (RT)-PCR

Total RNA was extracted from cells and tissues with Total RNA Kit I R6834 (OMEGA). The obtained RNA was subjected by using Moloney murine leukemia virus reverse transcriptase (M-MLV) (Takara) for specific reverse transcription of the positive and negative strands, respectively. The JEV positive-chain RNA was reverse transcribed using the JEV-Tag-R primer, and GAPDH-R, was amplified using the tag sequence as the reverse primer and JEV-F as the forward primer. The JEV negative-chain RNA was reverse transcribed synthesis using the JEV-Tag-F primer and GAPDH-R, was amplified using the tag sequence as the forward primer and JEV-R as the forward primer. The viral RNA levels were measured using a one-step real-time reverse transcription (RT)-PCR with SYBR Premix Ex Taq kit (TaKaRa), and the following primers were used (5′-3′): Tag sequence: TTTGCTAGCTTTAGGACCTACTATATCTACCT, JEV-Tag-F primer: TTTGCTAGCTTTAGGACCTACTATATCTACCTGGAATTTGAAGAGGCGCAC JEV-Tag-R primer: TTTGCTAGCTTTAGGACCTACTATATCTACCTGTACTCCACCACGATGGCTC, JEV-F primer: GGAATTTGAAGAGGCGCAC, JEV-R primer: GTACTCCACCACGATGGCTC, GAPDH-F: AAGGCCATCACCATCTTCCA, GAPDH-R: GCCAGTAGACTCCACAACATAC.

### *In vivo* Mouse Experiment

3-week-old female BALB/c mice were obtained from the Vitalriver Company in Beijing, housed in an environmentally controlled room, and maintained on standard laboratory food and water *ad libitum* throughout the study. To assess the virulence of the JEV mutant, intraperitoneal (i.p.) injections (10^6^ TCID_50_ /200 μL) and intracerebral (i.c.) injections (10^2^ TCID_50_ /30 μL) were used. Mouse survival and body weight were monitored daily. Then, three mice were randomly selected from each group after day 7 (i.p.) or 3 (i.c.) post-injection. Mice brain tissues were harvested separately, the virus solution was infected into BHK-21 cells, and brain virus titer was measured using the TCID_50_ method.

### Ethics Statement and Biosafety

All animal experiments involving recombinant JEV were reviewed and approved by the Institutional Animal Care and Use Committee at South China Agricultural University (SCAU) and were carried out in accordance with the approved guidelines.

### Statistical Analyses

Differences between experimental groups were determined using an unpaired *t*-test and analysis of variance (ANOVA) in GraphPad Prism software (GraphPad Software Inc.).

## Results

### The Pathogenicity *in vivo* and Growth Rate *in vitro* Are Compared Between SA14 and BJB Viruses

**Figure 1:**
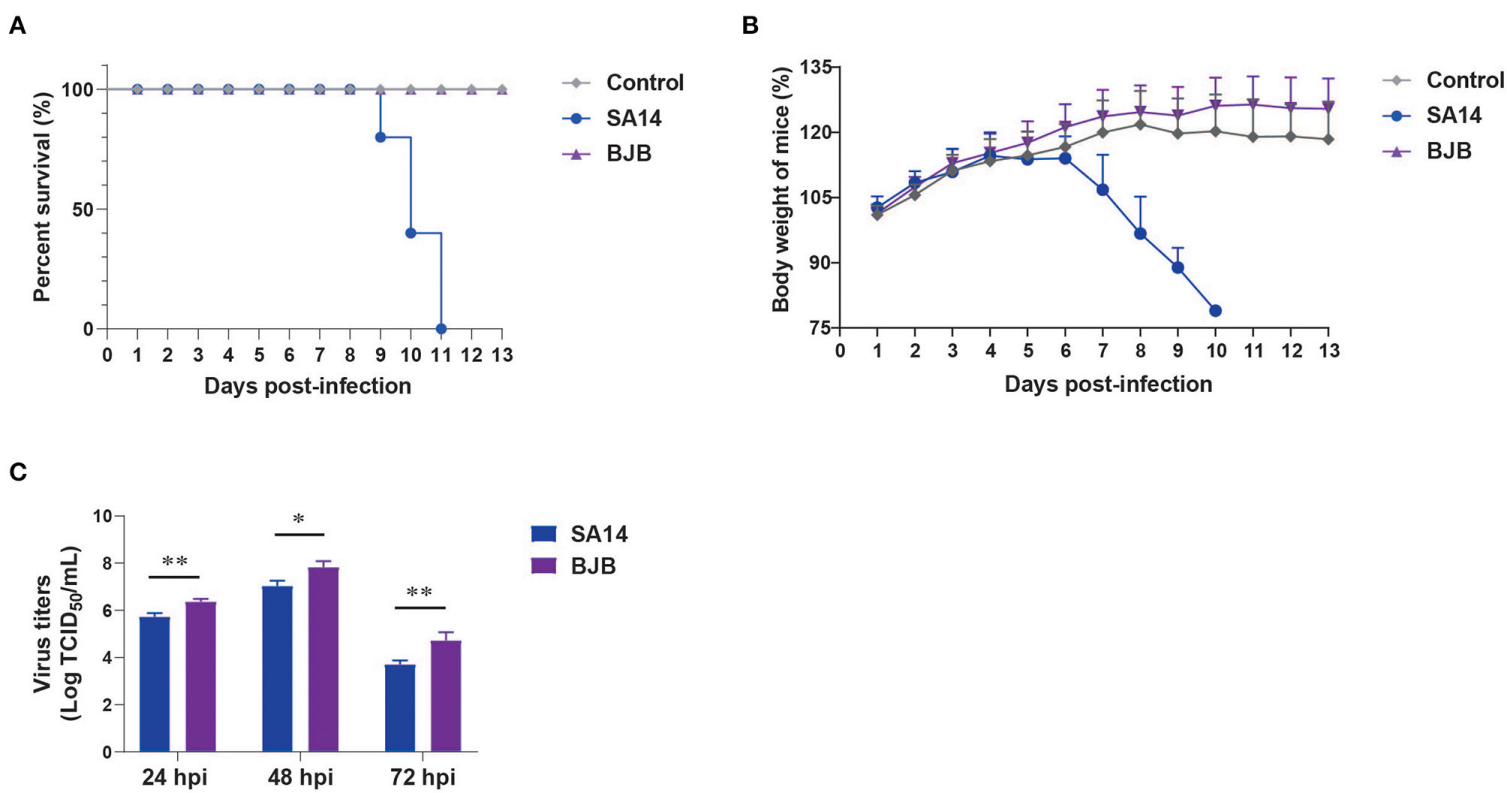
The pathogenicity and growth rate of SA14 and BJB viruses *in vivo* and *in vitro*. The SA14 and BJB viruses were injected intraperitoneal into SPF BALB/c (*n* = 5) mice using a dose of 10^6^ TCID_50_/200 μL. Survival rate **(A)** and body weight **(B)** were monitored daily for 13 days. Mice that lost more than 25% of their initial weight were euthanized. **(C)** BHK-21 cells seeded in 12-well plates were infected with SA14 or BJB viruses at a multiplicity of infection (MOI) of 0.001. Culture supernatant was then collected at 24, 48, and 72 hpi and subjected to TCID_50_ on BHK-21 cells. Data are shown as means ± SD (*n* = 3), and significance was calculated using an unpaired *t*-test (*P < 0.05; **P < 0.01).

To explore the pathogenicity of BJB strains isolated from humans, 3-week-old female BALB/c mice were infected with the SA14 and BJB virus strains using 10^6^ TCID_50_ by intraperitoneal (i.p.) injection. Mice infected with SA14 virus began to die from 9 days post-infection (dpi), and resulting in a 100% case fatality rate (Figure 1A). However, mice infected with BJB virus did not die, nor did they display obvious clinical symptoms or weight loss (Figures 1A,B).

*In vitro*, BHK-21 cells were infected with SA14 or BJB at a multiplicity of infection (MOI) of 0.001. Supernatants and cells were harvested at 24, 48, and 72 hpi. The viral titers were determined by TCID_50_. As shown in Figure 1C, the growth rate of BJB was higher than SA14 virus.

### The Nucleotide Mutations of 3′UTR Significantly Alter the RNA Secondary Structure of SL-IV and DB1 Structure Regions

The complete genome sequences for the SA14 and BJB virus strains were compared in order to identify nucleotides that consistently differ between these viruses. Through sequence analysis, some mutations at sites 248, 254, 258, and 307 were observed in the 3′UTR (Table 1). In order to explore the impact of these differences on viral characteristics, we used an infectious cDNA clone pACYC-SA14/U14163 as the parental JEV strain for engineering 3′UTR (T248C, A254G, A258G, and 307G). The parental virus SA14 (T248, A254, and A258) and recombinant virus rSA14-3′UTRmut (T248C, A254G, A258G and 307G) were rescued, biologically cloned, and sequenced in BHK-21 cells.

**Table 1:** Differences of nucleotide in the 3′UTR between SA14 and BJB.

	**Nucleotide position** ***^a^*** **in the 3′ UTR**			
**Virus**	**248**	**254**	**258**	**307**
SA14	T	A	A	-*^b^*
BJB	C	G	G	G

a *The nucleotide numbering refers to the start of the 3′UTR*.

b *“-” is indicated for a deletion of nucleotides*.

**Figure 2:**
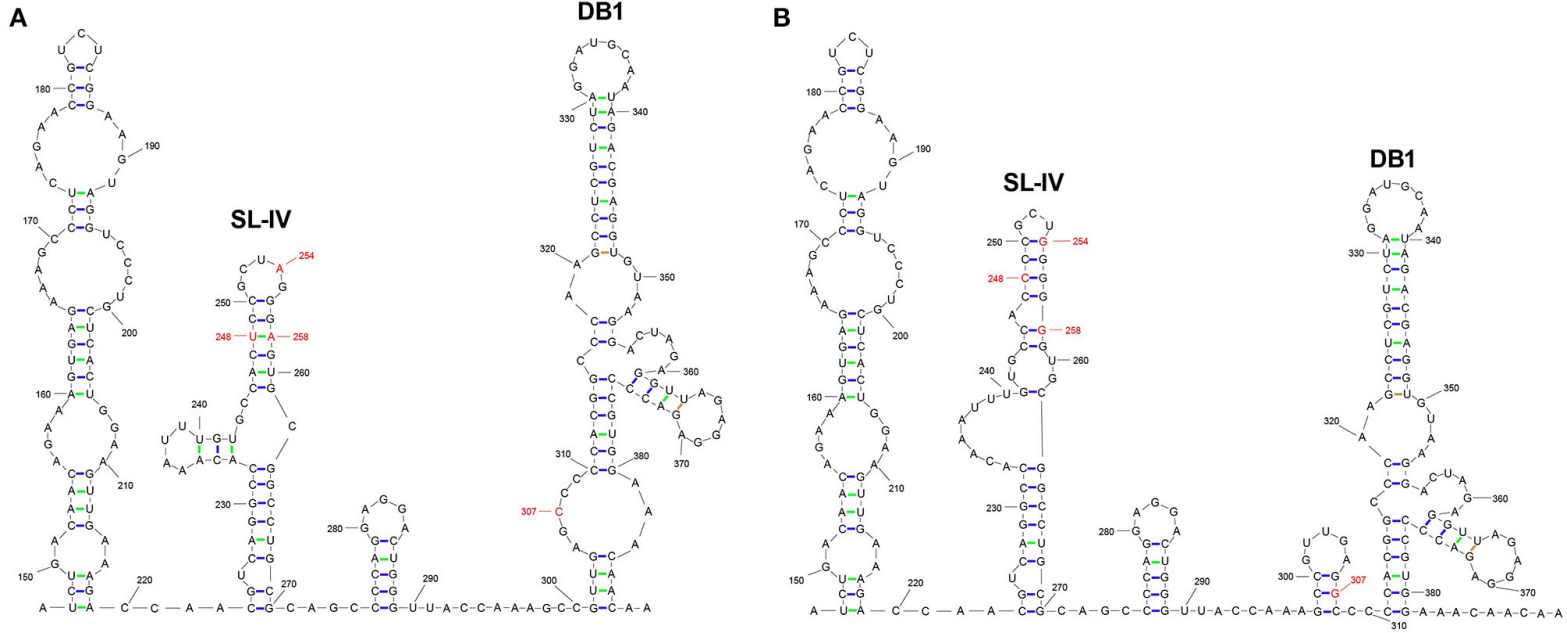
The prediction of RNA secondary structures in 3′UTR SL-IV and DB1 structure regions. The RNA secondary structures with the location of 3′UTR differences between JEV strains SA14 **(A)** and rSA14-3′UTRmut **(B)**. RNA structure was used to predict the RNAfold (http://rna.tbi.univie.ac.at//cgi-bin/RNAWebSuite/RNAfold.cgi) in the mutated regions.

Subsequently, we explored whether the mutation of 3′UTR would affect the secondary structure of RNA. RNAfold was performed structural prediction of the 3′UTR mutant region. According to the prediction of two viral 3′UTR secondary structures, the nucleotide sites 248, 254, and 258 were located in the SL-IV structure region, and the nucleotide site 307 was located in the DB1 structure region. The mutations of T248C, A254G, and A258G reduced the apiculus ring and increased the lateral ring significantly in the SL-IV structure region of 3′UTR (Figures 2A,B). Moreover, the insertion of 307G added a ring to the DB1 structure region. These differences suggested that the nucleotide mutations (T248C, A254G, A258G, and 307G) in 3′UTR significantly alter the RNA secondary structure SL-IV and DB1 regions.

### The Nucleotide Mutations of 3′UTR Promote Viral Growth Rate

**Figure 3:**
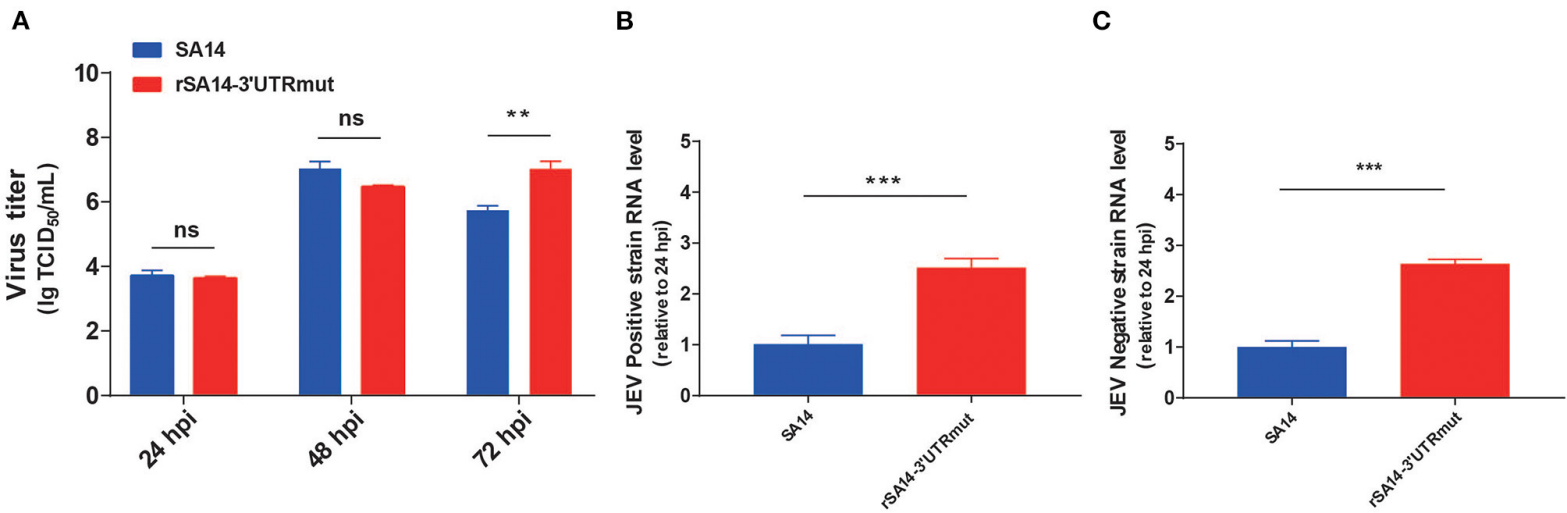
Virus replication of SA14 and rSA14-3′UTRmut. **(A)** BHK-21 cells seeded in 12-well plates were infected with rSA14-3′UTRmut or parental virus SA14 at a MOI of 0.001. Culture supernatant was then collected at 24, 48, and 72 hpi and subjected to TCID_50_ on BHK-21 cells. BHK-21 cells were infected with viruses at a MOI of 0.001, and then cells were harvested to extract total RNA. The obtained RNA was subjected to specific reverse transcription of the positive and negative strands, respectively. The viral positive strands **(B)** and negative strands **(C)** RNA levels were measured using a one-step real-time reverse transcription (RT)-PCR. Data are shown as means ± SD (*n* = 3), and significance was calculated using an unpaired *t*-test (^**^P < 0.01; ^***^P < 0.001).

To examine the growth properties of SA14 and rSA14-3′UTRmut *in vitro*, BHK-21 cells were infected with SA14 or rSA14-3′UTRmut at a MOI of 0.001. Supernatants and cells were harvested at 24, 48, and 72 hpi. The viral titers were determined by TCID_50_. As shown in Figure 3A, the titer of rSA14-3′UTRmut virus was higher than the parental virus SA14 by approximately 18-fold at 72 hpi. Subsequently, replication ability was compared by detecting the level of positive- and negative-chain RNA at 24 hpi, revealing that the positive- (Figure 3B) and negative-chain (Figure 3C) RNA levels of rSA14-3′UTRmut were higher than SA14 by approximately 2.5-fold. These results showed that the nucleotide mutations (T248C, A254G, A258G, and 307G) in 3′UTR promote viral replication.

### The Nucleotide Mutations of 3′UTR Reduce the Ability of Neuron Invasion

**Figure 4:**
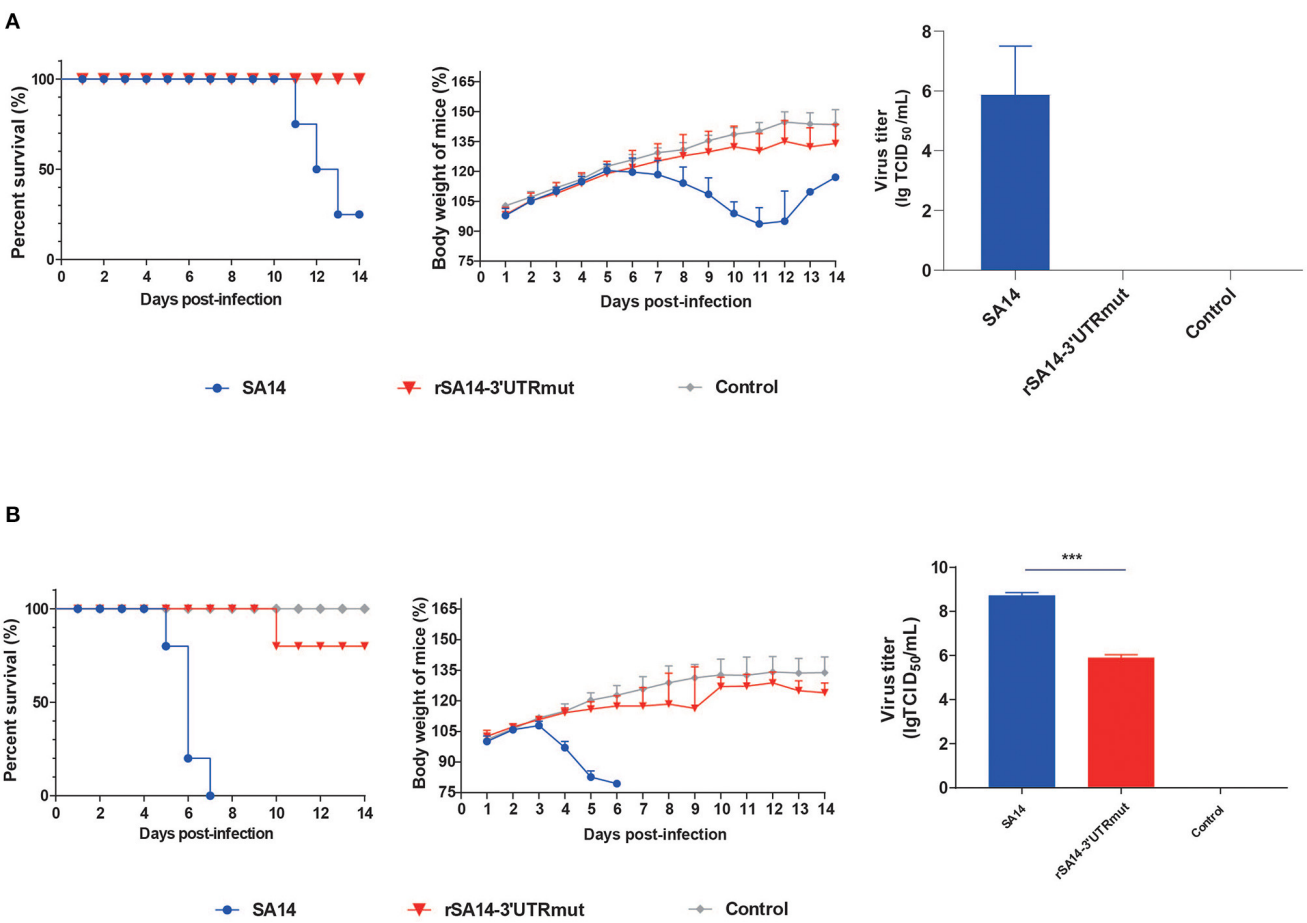
The neuron invasion and pathogenicity in mice. **(A)** The ability of the virus to invade neurons by the intraperitoneal inoculation route in mice. The SA14 and rSA14-3′UTRmut viruses were injected intraperitoneal into SPF BALB/c mice (*n* = 8) using a dose of 10^6^ TCID_50_/200 μL. Survival rate and body weight were monitored daily for 14 days. Then three mice were randomly selected from each group at day seven post-injection. Mouse brain tissues were harvested separately; the virus solution was infected into BHK-21 cells, and the brain virus titer was measured. **(B)** The pathogenicity of the virus by the intracranially inoculation route in mice. The SA14 and rSA14-3′UTRmut viruses were injected intracranially into SPF BALB/c mice (*n* = 8) using a dose of 10^2^ TCID_50_ /30 μL. Survival rate and body weight were monitored daily for 14 days. Then three mice were randomly selected from each group at day 3 post-injection. Mouse brain tissues were harvested separately; the virus solution was infected into BHK-21 cells, and the brain virus titer was measured. Mice that lost more than 25% of their initial weight were euthanized. The percentage weight from each group and each time point are presented as means ± SD (*n* = 3) and the significance was calculated using an unpaired *t*-test (^***^P < 0.001).

In order to investigate changes in the neural invasiveness of the virus in mice, 3-week-old female BALB/c mice were infected with the SA14 and rSA14-3′UTRmut viruses with 10^6^ TCID_50_ by i.p., injection. Mice infected with the SA14 virus died beginning at 11 dpi, showing signs of illness and weight loss (Figure 4A). Conversely, the rSA14-3′UTRmut virus was attenuated in mice, including only slight weight loss and no case fatality rate. Three mice were randomly selected from each group at 7 dpi to detect the viral burdens in the brain. The titer of SA14 in mice brains reached approximately 10^6^ TCID_50_. However, no virus was detected in mice brains infected with rSA14-3′UTRmut (Figure 4A). These results signified that the nucleotide mutations (T248C, A254G, A258G, and 307G) in 3′UTR reduce neuron invasion.

### The Nucleotide Mutations of 3′UTR Decrease Pathogenicity in Mice

As the above results proved that the nucleotide mutations (T248C, A254G, A258G, and 307G) in 3′UTR are responsible for neural tropism *in vivo*, we explored the effects of neural pathogenicity in mice. 3-week-old female BALB/c mice were infected with the SA14 and rSA14-3′UTRmut viruses with 10^2^ TCID_50_ by intracranially (i.c.) injection. SA14 was virulent in the mice, causing severe weight loss and 100% mortality (Figure 4B). However, the mortality rate of the rSA14-3′UTRmut virus reached just 20%, with only a slight weight loss. Three mice were randomly selected from each group at 3 dpi to detect the viral burdens in the brain. Strikingly, the rSA14-3′UTRmut virus decreased titers in mice brains by approximately 200-fold compared with SA14 in mice brains (Figure 4B). These results revealed that the nucleotide mutations (T248C, A254G, A258G, and 307G) in 3′UTR decrease pathogenicity in mice.

## Discussion

In China, before the 1970s and 1980s, the mainstream genotype was G-III. Around 1979, the G-I strains began to be introduced into China and gradually replaced G-III as the mainstream genotype (6, 7). Most existing vaccines in the world are G-III, which can completely protect against the G-III strain. However, studies have shown that the current G-III vaccine does not provide complete protection against the G-I strain, and the G-I vaccine also does not provide complete protection against the G-III strain (8, 9). Hence, the development of a new vaccine is particularly important. However, the requirements for the new vaccine include low virulence, high replication and strong immunogenicity. In this study, we compared pathogenicity between Japanese encephalitis virus SA14 and BJB (isolated from humans in the 1970s) strains, and we found that the BJB strain was attenuated in mice, with no case fatality rate. Strinkingly, the growth rate of BJB was higher than SA14.

Based on the sequence alignment of the viral genome between SA14 and BJB virus strains, some mutations at sites 248, 254, 258, as well as an insert at site 307 were observed in 3′UTR. The untranslated regions of flavivirus play a significant role in the virus life cycle. The study of RNA elements formed in the untranslated regions of other members of the Flaviviridae family (DENV, WNV, etc.) revealed the important functions in virus replication and significant impacts on lesion and mice pathogenicity (17, 29–31).

Subsequently, we predicte RNA secondary structure using RNAfold. The analysis showed that the nucleotide sites 248, 254, and 258 are located in the SL-IV structure region, and the nucleotide mutation 307 is located in the DB1 structure region. The nucleotide mutations T248C, A254G, and A258G cause the top loop of the SL-IV structure to shrink and the side loop to increase. The insertion of 307G adds a ring to the DB1 structure.

We became curious as to whether this secondary structure change affects the biological properties of the virus. We used an infectious cDNA clone (pACYC-SA14/U14163) as the parental JEV strain for engineering 3′UTR (T248C, A254G, A258G and 307G), and we rescued the mutant virus, which was named rSA14-3′UTRmut. The growth rate of the rSA14-3′UTRmut virus was higher than the parental virus SA14 at 72 hpi. However, there was no significant difference between 24 and 48 hpi. We speculated that the mutant virus would cause less cell lesions in the later growth period, which was conducive to the replication of the virus. The positive- and negative-chain RNA levels of rSA14-3′UTRmut were significantly higher than SA14 at 24 hpi. These results showed that the nucleotide mutations (T248C, A254G, A258G and 307G) in 3′UTR promote viral replication.

Subsequently, we investigated the changes in the neural invasiveness of the virus in mice. We found that the rSA14-3′UTRmut virus was attenuated in mice, including only slight weight loss and no case fatality rate, this compared to SA14, which caused death, illness and weight loss. The mice infected with SA14 started to die at 11 dpi not at 6 dpi as Figure 1A. We speculated that it might be due to individual differences, as well as differences in ambient temperature and humidity. Further, no virus was detected in mice brains infected with rSA14-3′UTRmut at 7 dpi. This indicated that the nucleotide mutations (T248C, A254G, A258G, and 307G) in 3′UTR reduce the ability of neuron invasion. We then explored the effects of the neural pathogenicity in mice. SA14 was virulent in mice, including severe weight loss and 100% mortality. However, the mortality rate of the rSA14-3′UTRmut virus reached just 20% with only slight weight loss. The rSA14-3′UTRmut virus decreased viral burdens in mice brains by approximately 200-fold compared with SA14. The neuron tropism of the rSA14-3′UTRmut virus in neuron cells *in vitro* needs further study. The reduction in mouse pathogenicity caused by changes in secondary structure may be due to SL-IV preventing Xrn1 from reducing the efficiency of viral RNA degradation and reducing sfRNA production, making the virus more susceptible to natural immunity and clearance (19, 32). The specific mechanism still needs further exploration and verification.

In summary, our study found that the SL-IV and DB1 regions of 3′UTR are essential for JEV replication, neural invasiveness, and viral pathogenicity. Our results confirm that the nucleotide mutations (T248C, A254G, A258G, and 307G) in 3′UTR can promote viral replication in BHK-21 cells, reduce the ability of neuron invasion, and decrease pathogenicity in mice. This study provides a new perspective for weakening candidate vaccine strains and increasing virus production.

## Data Availability Statement

The original contributions generated for this study are included in the article/supplementary material, further inquiries can be directed to the corresponding author/s.

## Ethics Statement

All animal experiments involving recombinant JEV were reviewed and approved by the Institutional Animal Care and Use Committee at South China Agricultural University (SCAU) and were carried out in accordance with the approved guidelines.

## Author Contributions

JX, YZ, WQ, and ML conceived and designed the experiments and wrote the manuscript. JX, YZ, ZL, LL, QX, and JL performed the experiments. JX, YZ, ZY, CH, and WQ analyzed the results of the experiments. All authors read and approved the final manuscript.

## Conflict of Interest

The authors declare that the research was conducted in the absence of any commercial or financial relationships that could be construed as a potential conflict of interest.

## Publisher's Note

All claims expressed in this article are solely those of the authors and do not necessarily represent those of their affiliated organizations, or those of the publisher, the editors and the reviewers. Any product that may be evaluated in this article, or claim that may be made by its manufacturer, is not guaranteed or endorsed by the publisher.
